# Absence of IL-10 production by human PBMCs co-cultivated with human cells expressing or secreting retroviral immunosuppressive domains

**DOI:** 10.1371/journal.pone.0200570

**Published:** 2018-07-12

**Authors:** Daniel Ivanusic, Heiko Pietsch, Jasper König, Joachim Denner

**Affiliations:** Robert Koch Institute, Berlin, Germany; Deutsches Primatenzentrum GmbH - Leibniz-Institut fur Primatenforschung, GERMANY

## Abstract

Immunosuppression by retroviruses including the human immunodeficiency virus—1 (HIV-1) is well known, however the mechanisms how retroviruses induce this immunosuppression is not fully investigated. It was shown that non-infectious retroviral particles as well as retroviral or recombinant retroviral transmembrane envelope (TM) proteins demonstrated immunosuppressive properties. The same was shown for peptides corresponding to a highly conserved domain in the TM protein. This domain is called immunosuppressive (ISU) domain and it induces modulation of the cytokine release of peripheral blood mononuclear cells (PBMCs) from healthy donors. In addition, it changes the gene expression of these cells. Common indications for the immunosuppressive activity were tumour growth *in vivo* and interleukin—10 (IL-10) release from human PBMCs *in vitro*. Single mutations in the ISU domain abrogated the immunosuppressive activity. In order to develop a new model system for the expression of the ISU domain and presentation to PBMCs which is not prone to possible endotoxin contaminations, two expression systems were developed. In the first system, designated pOUT, retroviral proteins containing the ISU domain were expressed and released into the cell culture medium, and in the second system, tANCHOR, the ISU domain was presented by a tetraspanin-anchored sequence on the cell surface of human cells. Both systems were exploited to express the wild-type (wt) ISU domains of HIV-1, of the porcine endogenous retrovirus (PERV) and of the murine leukaemia virus (MuLV) as well as to express mutants (mut) of these ISU domains. PERV is of special interest in the context of virus safety of xenotransplantation using pig organs. Expression of the TM proteins was demonstrated by confocal laser scanning microscopy, ELISA and Western blot analyses using specific antibodies. However, when cells expressing and releasing the ISU were co-incubated with human PBMCs, no increased production of IL-10 was observed when compared with the mutants. Similar results were obtained when the released TM proteins were concentrated by immunoprecipitation and added to PBMCs. We suggest that the absence of IL-10 induction can be explained by a low amount of protein, by the lack of a biologically active conformation or the absence of additional factors.

## Introduction

Retroviruses are like many other viruses including the measles virus (MV) and the human cytomegalovirus (HCMV) immunosuppressive. The mechanisms how viruses induce immunosuppression in the infected host are different and not fully elucidated. In the case of the MV, viral proteins effectively inhibit the induction of the innate immunity, especially the interferon production [1]. The persistence of the virus for many months indicates a prolonged immunosuppression and secondary infections occur because of it [2]. Measles-related symptoms are associated with lymphopenia and depletion of lymphocytes from lymphoid tissues [3]. MV proteins actively silence T cells by interfering with signalling pathways essential for T-cell activation and the MV glycoprotein complex was shown to activate cellular sphingomyelinases and to interfere with Akt kinase activation [4]. Suppressed immune responses in measles virus infections are also associated with an increased IL-10 production by neutrophils and monocytes may [5–7].

To coexist with its host, HCMV uses different mechanisms to enable the virus to remain invisible to cells of the immune system and to induce a general suppression of the immune system [8]. Viral proteins inhibit NK cell, helper T and cytotoxic T cell responses and target the humoral immune response. The virus also controls the production of cytokines and chemokines and stimulates local inflammation [9]. In addition, HCMV-encoded homologs of G protein-coupled receptors and chemokines play an important role in evasion from the immune system [10]. Furthermore, microRNAs regulate viral and cellular transcripts to favour viral infection and to inhibit the host`s antiviral immune response [11, 12].

**Fig 1:**
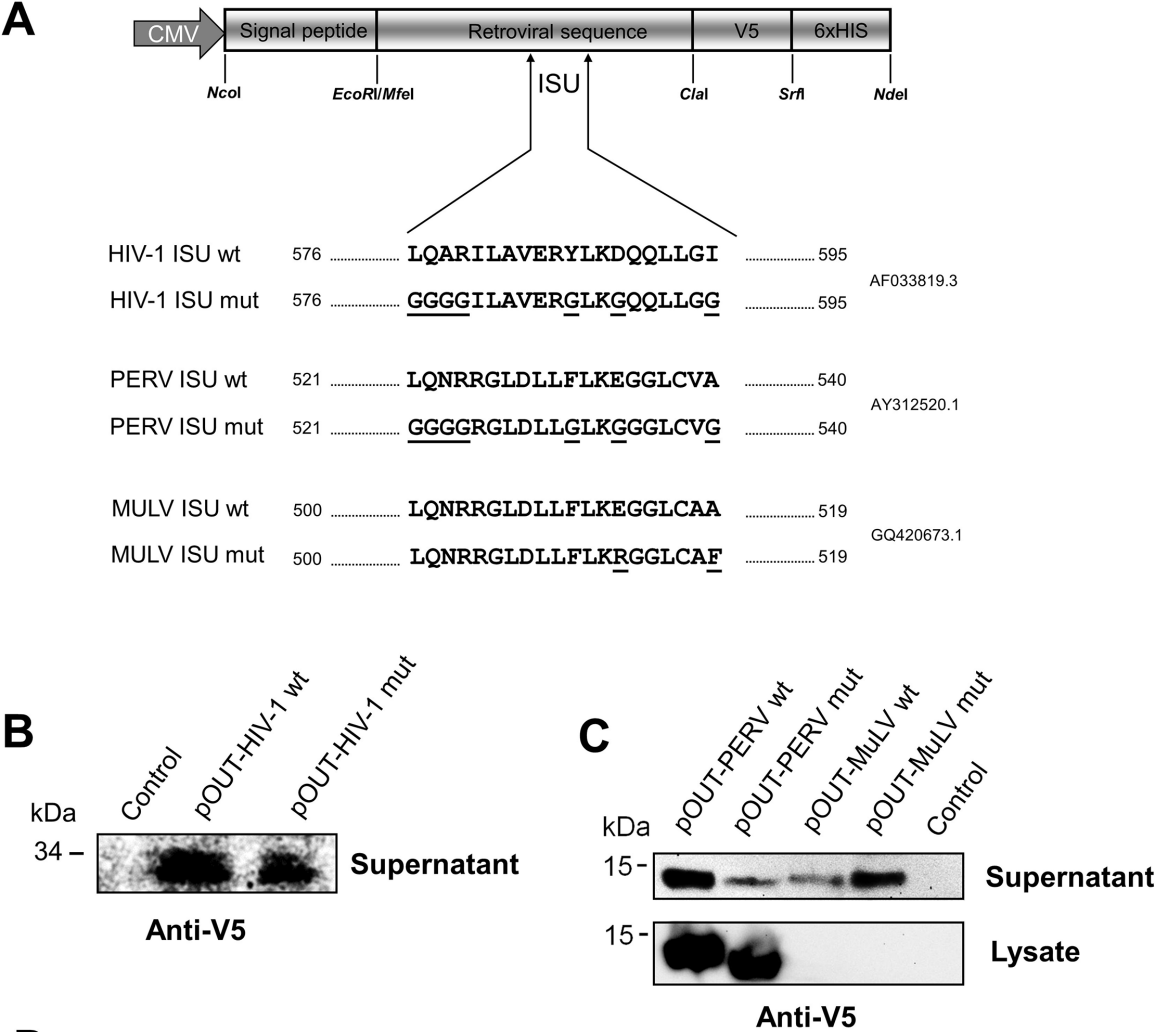
The pOUT expression system and analysis of the expressed proteins. **(A)** Schematic presentation of the vector and sequences of the corresponding ISU domains. Retroviral TM protein sequences containing the ISU domain are fused N-terminally to a secretion sequence (signal peptide) from the luciferase gene of *Gaussia princeps*, V5 and 6xHis tags are located in the C-terminus. The ISU domain protein sequences of HIV-1, PERV and MuLV and their mutated versions are shown. **(B)** Western blot analysis of the secreted His-tagged TM protein of HIV-1 and PERV containing the ISU domain. The proteins were concentrated by an His Tag Dynabeads pulldown, an anti-V5 antibody was used. **(C)** Western blot analysis of the His-tagged TM protein fragments in the supernatant and in the cell lysate of transfected cells using antibodies against the V5 tag. Proteins of PERV and MuLV with and without mutations in the ISU domain were analysed.

There is accumulating evidence that in the case of retroviruses also a viral protein, the transmembrane envelope (TM) protein, play a role in virus-induced immunosuppression [13, 14]. The human immunodeficiency virus type 1 (HIV-1) and HIV-2 as well as other retroviruses, e.g., gammaretroviruses such as the murine leukaemia virus (MuLV), the feline leukaemia virus (FeLV) and the koala retrovirus (KoRV) induce immunosuppression in the infected host (for review see [13, 14]). Non-infectious virus particles, purified viral or recombinant TM proteins were found immunosuppressive in different *in vitro* and *in vivo* assays. In mice, certain tumour cells grow to tumours in animals which are immunocompromised, but not in immunocompetent mice. Expression of TM proteins from different retroviruses on these cells allowed them to grow to tumours even in immunocompetent animals [15, 16]. This indicates that the TM proteins suppress the immune system und prevent tumour rejection. To localise the biologically active domain in the TM proteins, synthetic peptides were used and a domain in the C-terminal part of the N-helical repeat, the so-called immunosuppressive (ISU) domain was identified [17, 18] (Fig 1A). The ISU domain is highly conserved among retroviruses [14]. Synthetic 17- to 19-mer peptides corresponding to the ISU domain of gammaretroviruses or HIV-1 inhibited proliferation of PBMCs and modulated their cytokine release. For example, they caused an increase of IL-10 and had an inhibitory effect on protein kinase C (PKC) [14, 18–26]. Recombinant gp41 produced in bacteria [27–29] or human cells [30, 31] also modulated cytokine expression of PBMCs from healthy donors. Single mutations in the ISU domain abrogated the ability of retroviral ISU domains to cause IL-10 release and to modulate gene expression [30]. Single mutations also abrogated tumour growth *in vivo* in the murine experimental system described above [16, 32] and improved the efficacy of an antiretroviral vaccine [33]. Replication competent HIV-1 particles with such mutations in the ISU domain of gp41 did not induce IL-10 release, whereas the wild-type virus did [30].

Working with recombinant retroviral proteins produced in bacteria bears the risk that they are contaminated with endotoxin. Endotoxin induces similar, but not identical changes in the cytokine release by human PBMCs [31]. In addition, production of TM proteins in human cells and their release is not very effective and requires concentration of large amounts of culture fluid with the risk of endotoxin contamination. Therefore, here two new endotoxin-free expression systems for “one-pot protein expression and biological evaluation of IL-10 induction” were developed. In addition to the ISU domain of HIV-1, the ISU domain of PERV and MuLV were analysed. PERVs are of interest because these porcine viruses may be transmitted during xenotransplantation using pig cells, tissues or organs to the human recipient and may cause zoonotic infections [34]. MuLVs were the first retroviruses to prove that immunosppression occurs in its natural host species (for review see [13, 14]). The protein sequence of the ISU domain of PERV is identical to the sequences of ISU domains of FeLV, MuLV, and KoRV [14].

Retroviral TM proteins of HIV-1, PERV and MuLV are type I membrane proteins. They are anchored with a C-terminal membrane spanning domain (MSD) in the virus membrane. The TM protein gp41of HIV-1 is highly modified by N-linked glycans, but the p15E proteins of PERV, KoRV and MuLV are not glycosylated [35–37]. In order to study the immunosuppressive properties of the TM proteins, sequences of the TM proteins containing the ISU domain were selected. Two approaches were used. First, a cell system was established which highly efficiently secretes native retroviral TM proteins which were used to analyse their immunosuppressive properties. To obtain a high secretion rate, an efficient N-terminal signal peptide derived from the luciferase of the *Gaussia princeps* organism was used. This signal peptide was shown to allow a very effective secretion [38, 39]. Fusion of this signal peptide to retroviral proteins facilitated secretion of these proteins into the culture medium of human producer cells. The second approach was based on the surface expression of a part of the TM protein containing the ISU domain using the tetraspanin CD82.

## Results

### Expression of the ISU domains in the pOUT system

**Fig 2:**
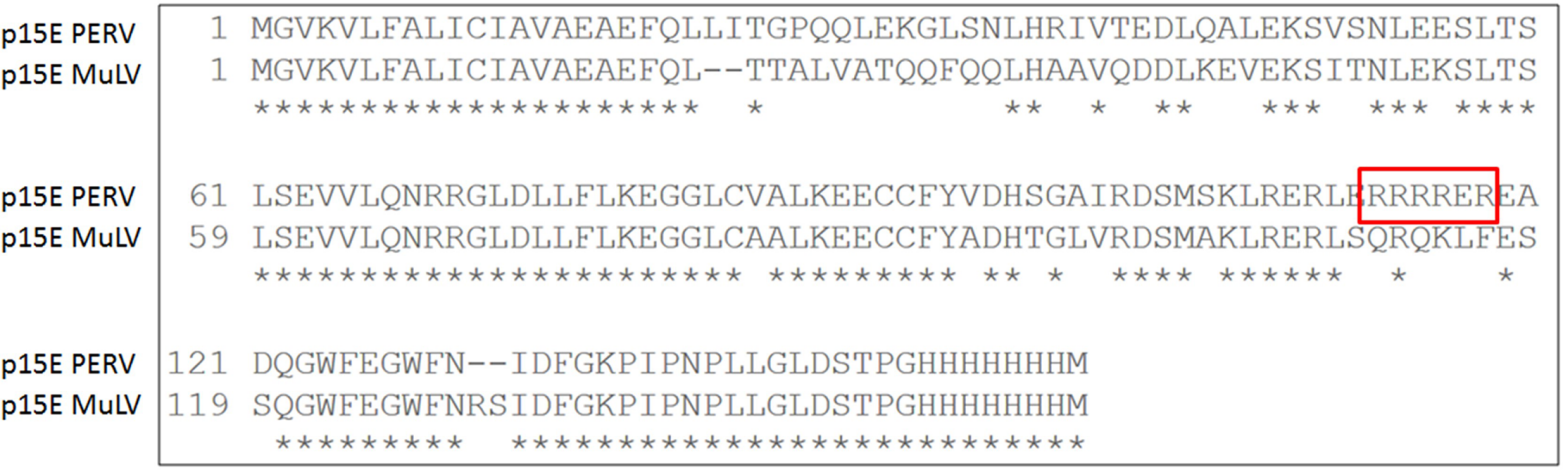
Sequence alignment of the TM proteins p15E of PERV (GenBank AY312520.1) and MuLV (GenBank GQ420673.1). The arginine rich sequence in the p15E of PERV is framed in red.

Using the newly developed pOUT system, the TM proteins of HIV-1, PERV and MuLV and their mutants were expressed in HeLa or HEK293T cells and secreted into the supernatant. The extracellular parts of the TM proteins containing the ISU domain and C-terminal V5 and 6xHis tag sequences were expressed under the control of the CMV promoter (see Materials and methods) (Fig 1A and S1 Fig). For a high secretion performance a signal peptide derived from the Gaussia luciferase gene (*Gaussia princeps)* was used [38]. Efficient secretion was shown for the wt ISU domains of HIV-1, PERV and MuLV and the corresponding mutants (mut) by Western blot analysis using antibodies against the V5 tag (Fig 1B and 1C). The expression of the ISU domain of PERV was—in contrast to that of MuLV—observed mainly intracellular (Fig 1C). Comparing the sequences of the TM proteins of both viruses the only difference was an arginine repeat in the protein sequence of p15E of PERV (Fig 2). The short arginine repeat suggests that this protein was retained in the cells, because arginine/serine rich proteins are mainly localised in the cytoplasm and are targeted to the nucleus [40, 41].

**Fig 3:**
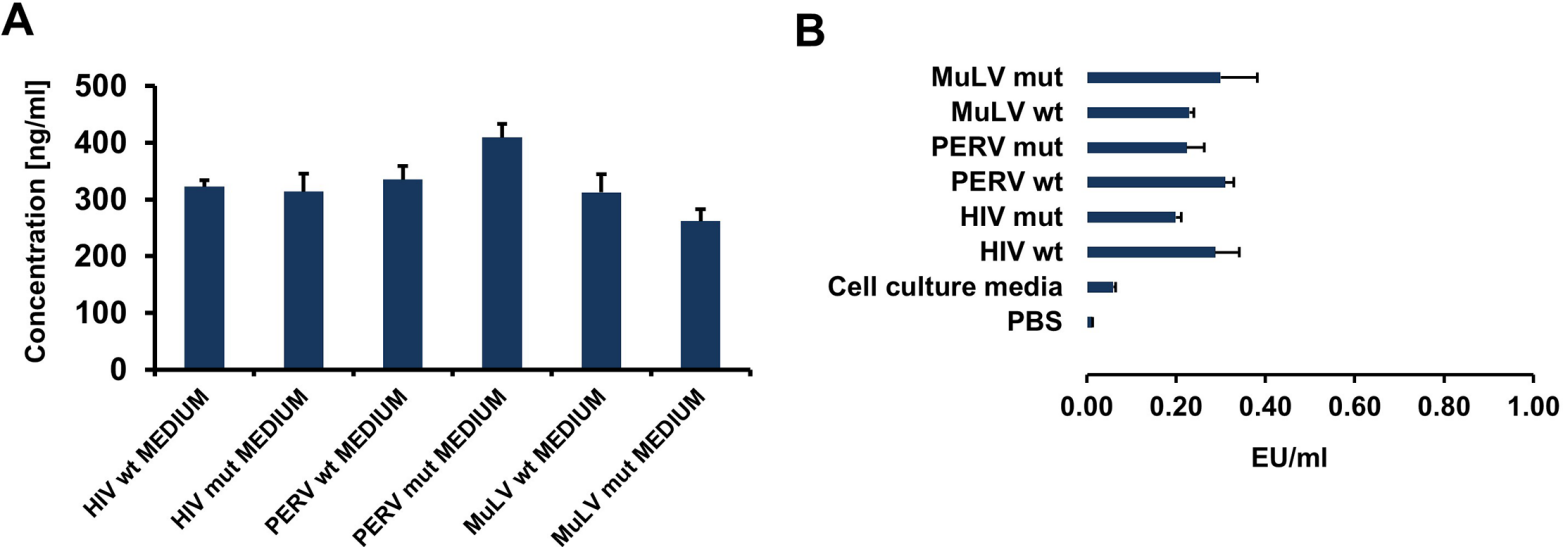
Characterisation of the concentrated TM proteins of HIV-1, PERV, and MuLV. **(A)** Quantification of the TM proteins using an ELISA-based method. Ni(2+) chelate-coated 96 well plates are used to quantify 50 μl of batches which are 40 x concentrates of secreted TM proteins. To quantify the concentration of collected and secreted TM proteins a standard curve of highly purified polyhistidine tagged gB protein from HHV-6 was used (S2 Fig) **(B)** Results of LAL testing using 50 μl concentrated supernatants, samples were measured in duplicates.

In order to quantify the amount of ISU proteins in the supernatant of pOUT protein-releasing cells, an ELISA was performed using NTA-Ni(^2+^) chelate-coated plates. The collected and concentrated supernatants from cells secreting proteins containing sequences of wt HIV, mut HIV, wt PERV and mut PERV were added to the wells of the plates, detected by an HRP conjugated anti-His antibody and the absorbance was measured at 450 nm. Using a standard titration curve of the HIS-tagged glycoprotein B (gB) from the human herpesvirus 6 (HHV-6), which was produced in our laboratory in highly purified quality [42] (S2 Fig), the protein amount was estimated, between 262 and 410 ng/ml were found secreted (Fig 3A). When the concentrated protein samples were analysed using the LAL assay, a contamination with a low amount of LPS (ranging from 0.2–0.3 EU/ml) was observed (Fig 3B).

### Expression of the ISU domains in the tANCHOR system

**Fig 4:**
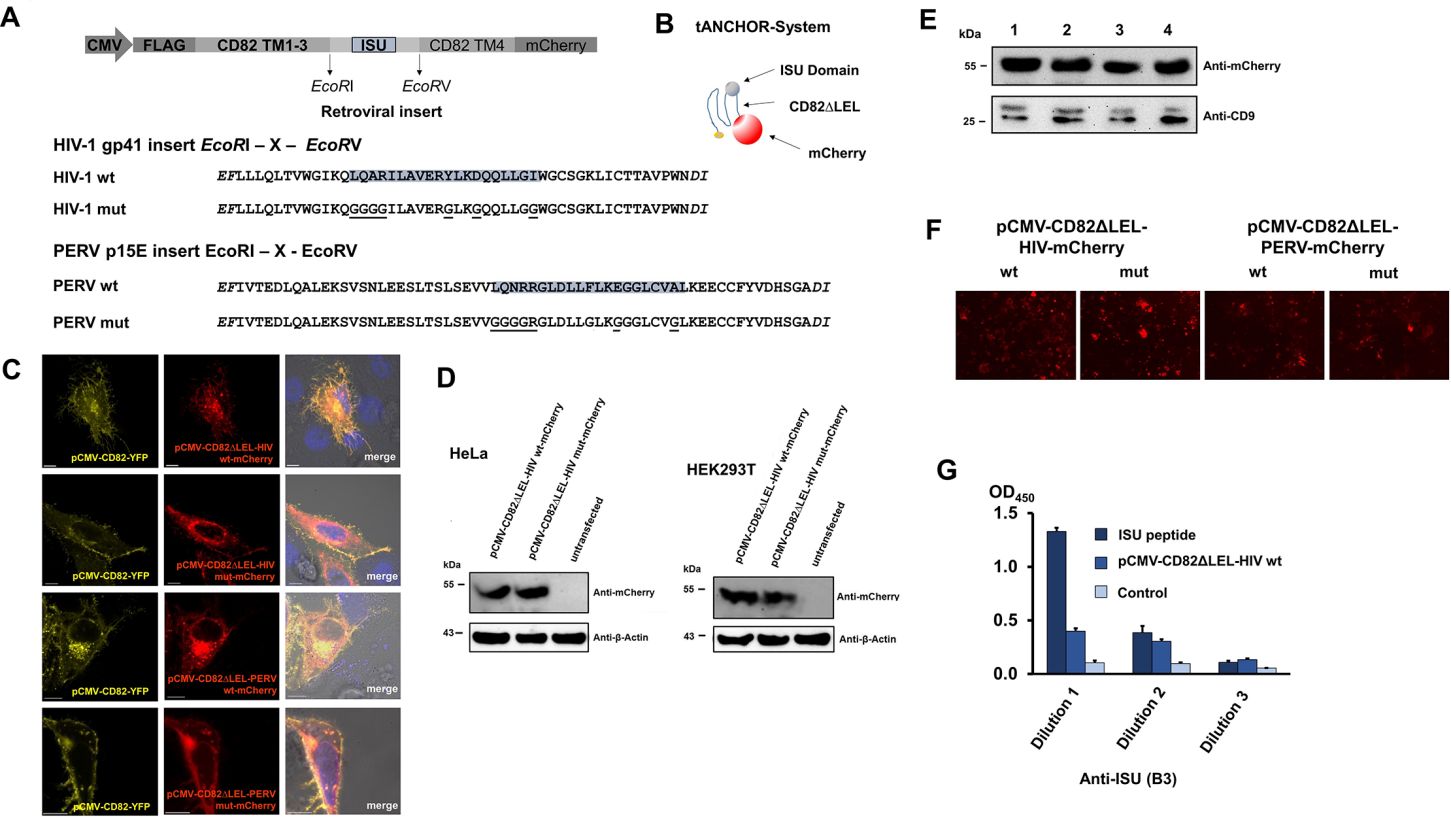
The tANCHOR expression elements and protein expression analysis. **(A)** Schematic presentation of the main expression elements. Retroviral TM sequences containing the ISU domain are fused N-terminally to a FLAG tag and the CD82 TM1-3 sequences and C-terminally to the CD82TM4 and mCherry sequences. The protein sequences of the HIV-1 and PERV TM part containing the ISU domain (framed or marked grey) and the corresponding mutants (mutations are underlined) are shown. **(B)** Schematic presentation of the tANCHOR membrane spanning proteins. The constructs contain the ISU domain instead of the LEL of CD82 (CD82ΔLEL). The FLAG tag is shown in yellow, the mCherry protein in red. **(C)** Confocal laser scanning microscopy images of HeLa cells expressing the CD82 anchored retroviral protein sequences tagged C-terminally with mCherry. In parallel a vector expressing the full-length protein sequence of CD82 under a CMV promoter and fused to YFP was used as a control. The merged image shows a similar cellular localisation. **(D)** Comparison of the protein expression in human cell-lines. Western blot analysis of the expressed CD82 anchored retroviral protein sequences in HeLa and HEK293T cells using anti-mCherry antibodies. Antibodies against beta actin were used as loading control. **(E)** Analysis of the protein expression level on cells used in these experiments. HeLa cells are transfected with the vectors (1) pCMV-CD82ΔLEL-HIV-WT, (2) pCMV-CD82ΔLEL-HIV-MUT, (3) pCMV-CD82ΔLEL-PERV-WT and (4) pCMV-CD82ΔLEL-PERV-MUT, cell lysates are analysed by Western blot analysis using anti-mCherry antibody. Detection of CD9 using specific antibodies was used as loading control. **(F)** Protein expression analysis by detection of the mCherry fluorescence in 96 well plates. **(G)** Detection of the ISU domain expressed on the surface of cells expressing the HIV-1 tANCHOR construct. A cell-based and a peptide-based ELISA were performed using a purified IgG preparation of a monoclonal antibody specific for the ISU domain, synthetic ISU peptide was used as positive control, non-transfected cells were used as negative control.

In the tANCHOR expression system, the ISU domain was included into the sequence of the tetraspanin CD82, substituting the second extracellular loop (EC2, large external loop, LEL). The construct contained a FLAG tag and a mCherry sequence as a reporter protein (Fig 4A and 4B). The CD82-anchored and mCherry-tagged retroviral TM proteins were expressed in HeLa cells and confocal laser scanning microscopy analysis revealed that the expressed proteins are localised mainly in the plasma membrane. The co-localisation with the wild-type protein CD82 fused to YFP demonstrated that they are predominantly expressed on the cell surface with an extracellular orientation (Fig 4C). The rate of expression of the wild-type and mutated retroviral proteins was identical as demonstrated by Western blot analysis (Fig 4D). Further, the transfection efficiency of every performed transfection was comparable (S3 Fig). Expression of the wt and the mut proteins were approximately identical when the expression in HeLa cells was analysed by Western blot (Fig 4E).

In order to investigate the extracellular accessibility of the expressed ISU domain, binding of the monoclonal antibody B3, which is specific for the ISU domain of HIV-1, was analysed by a cell-based ELISA technique. The monoclonal antibody has been obtained by immunisation of a mouse with the synthetic ISU peptide conjugated to bovine serum albumin (BSA) and selection by an ELISA using immobilised ISU peptide, the epitope ILAVERYL was determined using overlapping peptides (Andreas Hoffmann, Joachim Denner, Paul Ehrlich Institute, Langen, unpublished data). Purified IgG were used in this cell-based ELISA. Binding of the ISU-specific IgG to the synthetic ISU peptide, and to cells expressing the wt ISU domain of HIV-1, but not to control cells was observed (Fig 4G).

### Influence of the immunosuppressive domains on cytokine release

**Fig 5:**
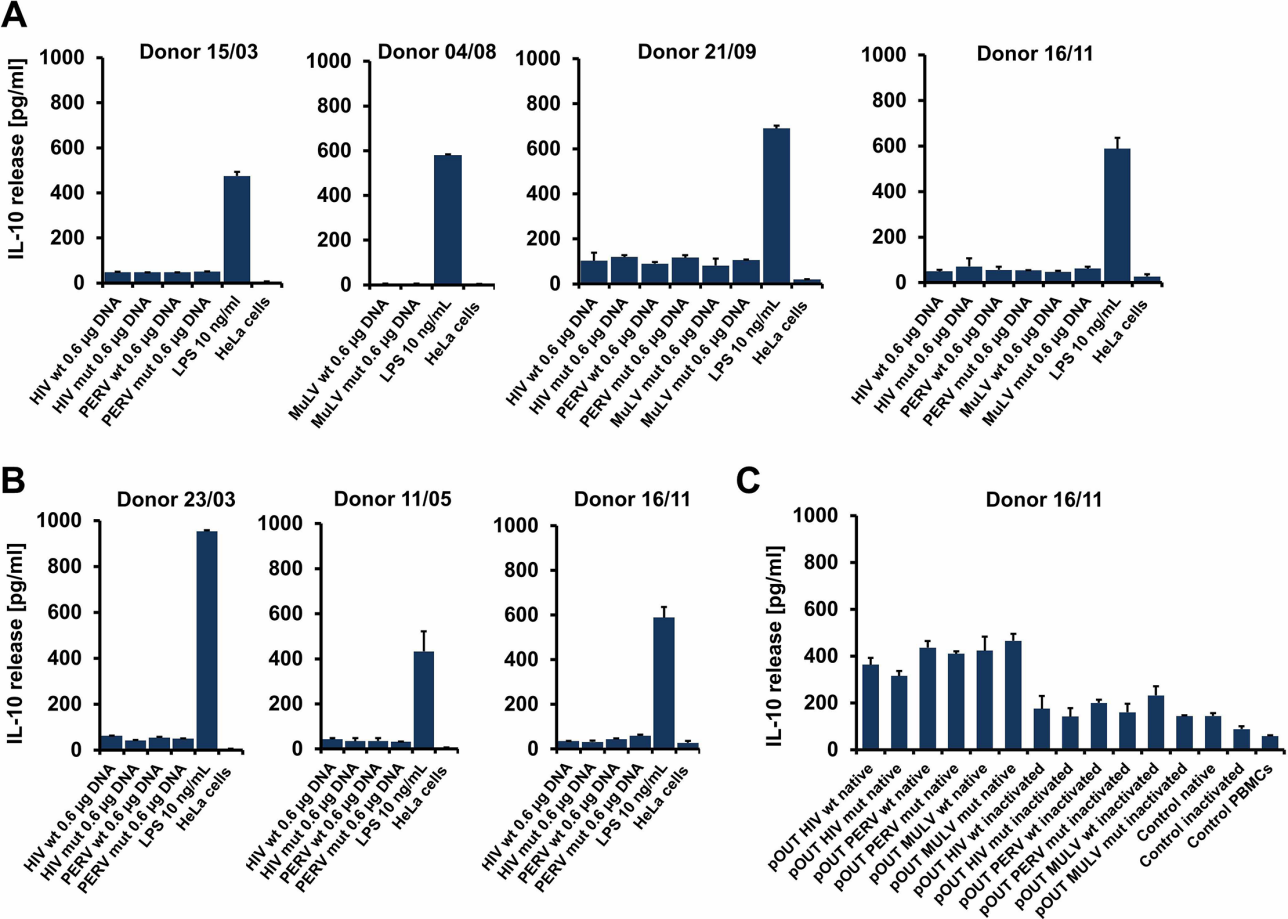
Analysis of IL-10 release after incubation of cells expressing the pOUT and tANCHOR constructs. **(A)** HeLa cells transfected with an indicated amount of DNA encoding the pOUT constructs WT and MUT HIV, WT and MUT PERV were incubated with human PBMCs from four different donors (the number indicate day/month of the experiment) and the IL-10 release was measured using an IL-10 ELISA. Different concentrations of LPS added to the cells were used as positive control, non-transfected HeLa cells were used as negative control. The error bars mean SD. The calculated p-values for the difference between the IL-10 release pf PBMCs incubated with untreated HeLa cells or with HeLa cells expressing pOUT with wt oder mut sequences was 5.93E-11. **(B)** The same for the pOUT constructs WT and MUT MuLV and PBMCs from three different donors. **(C)** IL-10 release induced by concentrated supernatants (40x) collected from HEK293T cells transfected with the pOUT constructs. Each well was incubated with 50 μl 40x concentrated supernatant before and after heat treatment. The p-values for the difference between the heat-inactivated supernatant and the untreated material were 0.0082 for HIV wt and mut, 0.067 for MuLV wt and mut, and 0.026 for PERV wt and mut. Concentrated supernatant from non-transfected 293 cells (native and heat inactivated) as well as PBMCs alone were used as control.

To analyse whether the ISU domains are able to induce expression of IL-10 *in vitro*, in human PBMCs, first, cells expressing the tANCHOR and the pOUT constructs were co-incubated with human PBMCs. Second, concentrated supernatants of cells expressing the pOUT constructs were added to human PBMCs, and in both cases the release of IL-10 was measured. When cells, which were transfected with different concentrations of pOUT constructs encoding for the ISU domains of HIV-1 or PERV were co-incubated with human PBMCs, low amounts of IL-10 were released and no significant differences between the wildtype and mutated proteins were observed. Although the amount of released IL-10 was higher compared with the amount released after incubation of human PBMCs with the supernatant from non-transfected HeLa cells (p = 5.93E-12) (Fig 5A), the constructs expressing the wild-type and the mutated versions of the ISU domain of HIV-1 and PERV gave the same results. The same was observed when cells expressing the tANCHOR constructs were incubated with human PBMCs (p = 5.81E-12) (Fig 5B). Altogether experiments with six different donors were performed (Fig 5A and 5B). In all cases lipopolysaccharide (LPS) served as a positive control inducing concentration-dependent high amounts of IL-10 (Fig 5A and 5B). No IL-10 was released when the wt and the mutant of the ISU domain of MuLV were expressed in HeLa cells and these cells were co-incubated with human PBMCs (Fig 5A, Donor 04/08). Notably, heat inactivation of the concentrated supernatants allowed to differentiate between protein based and heat-resistant, possible LPS-based IL-10 induction. The decrease of IL-10 release after heat treatment indicates the presence of a protein able to induce IL-10, however no differences in IL-10 production levels between wt and mut protein samples were observed (Fig 5C).

As we had been shown previously, PBMCs from different donors reacted differently to identical batches of ISU peptide homopolymers [26]. To make sure that the negative result, e.g., absence of increased IL-10 release after incubation of human PBMCs with ISU-expressing cells, is not due to a low-responder donor, the experiment was repeated with PBMCs of five other donors, in all cases the result was the same (Fig 5 and S4 Fig). Differences in the IL-10 release after incubation with LPS indicated differences in responding of different PBMCs (Panel A in S4 Fig). In addition, an experiment was performed adding increasing amounts of plasmid (Panel C and D in S4 Fig). Increasing amounts of plasmid were not associated with an increase in the IL-10 release, this indicates, that the plasmids used for transfection were not contaminated with residual LPS after the purification and washing procedure.

## Discussion

Here for the first time two new expression vector systems were designed and used to express the ISU domain of three different retroviruses and to present them to human PBMCs in order to study their ability to induce IL-10 as marker of immunosuppression. The first expression system is based on the expression and release of parts of the TM proteins containing the ISU domain (pOUT) (Fig 1), the second on the expression of the ISU domain using a CD82 anchor (tANCHOR) (Fig 4). In this study two gammaretroviruses (PERV, MuLV) and one lentivirus, HIV-1, were analysed. The sequences of the ISU domains of gammaretroviruses, betaretroviruses and lentiviruses are related, but not identical [14]. The sequences of the ISU domains of the gammaretroviruses MuLV, PERV, KoRV and FeLV are 100% identical [14], however there are sequence differences in the N and C terminal regions (Fig 2). Although a significant IL-10 release as a marker of an immunosuppressive activity was observed when human PBMCs were co-incubated with cells expressing and releasing (pOUT) or presenting the CD82 anchored ISU domain (tANCHOR), the same level of IL-10 release was observed with cells expressing the mutated ISU domains (Fig 5 and S4 Fig). This means that either the mutation did not inactivate the IL-10-inducing activity of the ISU domain or another heat-labile protein is biological active.

The need for an “one-pot protein expression and IL-10 validation system” secreting proteins into the supernatant or presenting proteins on the surface of human cells emerged because LPS is also able to induce IL-10 and possible contaminations in other assay systems could not be excluded. It was shown in the past that primary human immune cells are highly sensitive to LPS and can be activated by LPS concentrations as low as 0.02 ng/ml [43]. Particularly, the fact that protein concentration procedures may be associated with increased LPS concentrations [44] made it necessary to avoid experimental designs were proteins have to be concentrated. All reagents in the laboratory are associated with the potential risk of an endotoxin contamination. During previous experiments using recombinant gp41 produced in human cells and including methods to remove endotoxin, differences in the induction of cytokines by gp41 and LPS have been observed. For example proteins encoded by the genes serpine E1, IL1Ra, uPAR, PDGF-AA, thrombospondin-1, myeoloperoxidase and osteopontin have been induced by gp41 containing the ISU domain, but not by the LPS used [31]. However, under certain conditions these proteins have also been induced by defined LPS batches [45–51]. Furthermore, lipopolysaccharides from distinct pathogens induce different classes of immune responses *in vivo* [52]. However, when the concentrated samples were heat-inactivated, a significant loss of activity was observed despite the fact, that residual IL-10 induction is possibly due to a heat-resistant endotoxin (Fig 5C). LPS is stable at the temperature of 98°C used [53]. Although the loss indicates a protein as source, no differences between the activities of the wild-type or mutated ISU domain were observed. As mentioned above, either the mutations used did not inactivate the IL-10 inducing activity of the ISU domain or another protein was involved.

The data presented here are consistent with our previous results showing an absence of IL-10 release after incubation of human PBMCs with human melanoma cells expressing the TM protein of HERV-K, with human cells producing PERV and with human cells expressing the TM protein gp41 of HIV-1 [54]. The data are also in line with observations by Hummel et al. [55], showing that the Env proteins containing the ISU domain from three HERVs (syncytin-1, which is the Env of HERV-W; syncytin-2, which is the Env of HERV-FRD and the Env of HERV-K) expressed as recombinant proteins on the surface of Chinese hamster ovarial (CHO) cells did not induce IL-10. In contrast, the human choriocarcinoma cells naturally expressing these three proteins, syncytin-1, syncytin-2, and the Env protein of HERV-K on their surface, induced IL-10 expression by dendritic cells [55]. These data suggest that the amount of proteins expressed after transfection, or their conformation is not sufficient to interact with a putative receptor and to induce IL-10 [54, 55]. Especially in the our case of the ISU domain expressed in CD82 with its four membrane-spanning domains it may be expected that larger neighbouring proteins are the reason of steric hindrance. We cannot exclude that additional factors expressed in human choriocarcinoma cells may interfere with the Env proteins and trigger the immunosuppressive activity.

The absence of IL-10 induction after incubation of cells expressing and releasing the ISU domain reported here and previously [54, 55] is in contrast to numerous results obtained with synthetic peptides corresponding to the ISU domain or TM proteins of different retroviruses including HIV-1 (for review see [14]). Synthetic peptides corresponding to the ISU domain of HIV-1 conjugated to BSA [17, 18, 20] or in the form of homopolymers [26], had been shown to inhibited proliferation of human PBMCs. The conformation of the ISU domains induced by conjugation to a carrier protein or homopolymerisation seems to be important for the biological activity since ISU peptide monomers did not show immunosuppressive properties. Recombinant or purified viral TM proteins from different retroviruses such as HIV-1 [27–31], HERV-K [56], and FeLV [57–60] also inhibited proliferation of PBMCs and modulated cytokine release including an increase in the release of IL-10, whereas for example the Gag protein of FeLV was inactive. Non-infectious virus particles of PERV [56], FeLV [60–62], KoRV [63], HERV-K [56] and HIV-1 [30] were also able to inhibit mitogen-triggered lymphocyte proliferation, and to modulate cytokine release and gene expression. On the other hand it cannot be excluded that in some experimental settings using purified virus preparations or concentrated recombinant proteins these might have been contaminated by microbial components such as LPS. However, this seems highly unlikely in the cases mutants of the ISU domain have been compared with the wild-type ISU domain *in vitro* [14, 30] and *in vivo*, in mice [16, 32, 33].

The mechanism how the ISU domain interacts with immune cells is still unclear. There are several reports showing binding of the TM proteins or synthetic peptides corresponding to the ISU domain to monocytes and B cells [64–68].

It is well known that infection of natural hosts, e.g., sooty mangabeys with simian immunodeficiency viruses (SIV) is non-pathogenic despite high viremia [69]. SIV contain a functional ISU domain and may induce immunodeficiencies in non-natural hosts, e.g., rhesus monkeys. As proposed earlier, if the ISU domain plays a substantial role in the induction of the immunodeficiency, their receptor or the corresponding signal transduction should be non-functional in the natural host [14]. Interestingly, recently a 17-amino acid longer sequence of the toll-like receptor 4 (TLR-4) associated with a reduced response to TLR-4 ligands including LPS was found in all natural hosts of SIV, but not in non-natural host [70]. It is important to learn how the longer TLR-4 and other genetic changes in natural hosts prevent the outbreak of the immunodeficiency.

To summarise, the immunosuppressive domain is highly conserved among different retroviral genera. Synthetic peptides corresponding to this domain, recombinant and viral transmembrane envelope proteins and non-infectious particles of several retroviruses including HIV-1 have been shown to have an immunosuppressive activity, modulating the cytokine release of normal human PBMCs and modulating the gene expression in these cells [14, 26] (Table 1). Tumour cells not growing to tumours in immunocompetent mice grow to tumours when expressing the transmembrane protein of different retroviruses, indicating the immunosuppressive activity of these proteins [15, 16, 32]. Single mutations in the ISU domain abrogated their immunosuppressive activity [16, 30, 32]. In contrast, the expressed TM proteins of PERV, MuLV and HIV-1 described here as well as cells expressing the ISU domains of three HERV (W, FRD, K) [54, 55] were unable to induce IL-10 for reasons still to be elucidated. One conclusion from these investigations is the consideration, not to use only IL-10 measurement as a marker for an immunosuppressive property since LPS may always interfere. Therefore other assays and analysis platforms should be established to measure immunosuppressive activities of retroviral proteins. In addition, efforts should be undertaken to present the ISU domains in a conformation and/or multiplicity, e.g., containing multiple ligands to interact with a complex receptor, as found in the retrovirus infected individual [14].

**Table 1:** Differences in the immunosuppressive properties of retroviral TM proteins.

Retroviral TM proteins showing immunosuppression	Retroviral TM proteins NOT showing immunosuppression
Three HERV Env proteins^a)^ naturally expressed on human choriocarcinoma cells [55]	Three HERV Env proteins^a)^ expressed as recombinant proteins on CHO cells [55]
Retroviral TM proteins^b)^ expressed in a tumour cell rejection model [15, 16, 32]	HERV-K Env protein naturally expressed on teratocarcinoma cells [54]
Numerous purified viruses, viral TM proteins and peptides corresponding to the isu domain [13, 14, 17–33, 56–60]	ISU domains of HIV-1, PERV, and MulV, presented by the pOUT or tANCHOR systems on human cells (this study)

^a)^Syncytin-1, which is the envelope protein of HERV-W, syncytin-2, that of HERV-FRD, and the Env protein of HERV-K (HML-2)

^b)^Murine gammaretrovirus, type D retrovirus, HERV-H, ERV3, HERV-p(B9), HERV-V, syncytin-1 (HERV-W), syncytin-2 (HERV-FRD), syncytin-A, syncytin-B

## Materials and methods

### Cloning of expression constructs

Two expression systems were developed, one expressing and secreting the TM protein containing the ISU domain, designated pOUT expression constructs, the other expressing part of the TM protein including the ISU domain on the cell surface, using the CD82 tetraspanin, designated the tANCHOR system.

The pOUT expression constructs were generated by using the gene synthesis fragments SEQ ID 01 and 02 which represents the wild-type (wt) and mutated (mut) ectodomain of the transmembrane envelope protein gp41 of HIV-1 (aa 535–573, Genbank accession no. JQ975395.1, S1 Fig), SEQ ID 3 and 4 the wt and mut ectodomain of p15E of PERV (aa 477–585, Genbank accession no. AY312520.1), and SEQ ID 5 and 6 the wt and mut ectodomain of p15E of the Friend-MuLV (aa 458–566, Genbank accession no. GQ420673.1). The sequences including the ISU domain or the mutants in the ISU domain (Fig 2A) were cloned using the flanking *Nde*I/*Nco*I restriction sites in the vector pA4E211-FlexTEC (ATG:biosynthetics, Freiburg, Germany). These constructs contain a signal peptide derived from *Gaussia princeps* (Fig 2A), for improving the secretion efficiency [34]. The pA4E211-FlexTEC vector (ATG:biosynthetics, Merzhausen, Germany) contained a *puromycin* resistance marker.

The tANCHOR system was used to express retroviral TM proteins on the surface and constructed using the pCMVtag2B vector (Stratagene, Heidelberg, Germany) as backbone. The tetraspanin CD82 sequence (codons 1–267, accession no. NM_002231.3) was introduced instead of the CD63 sequence into the vector pCMV-CD63-YFP-FLAG [63] using the restriction sites *Not*I/*Xho*I and the synthetic gene fragment SEQ ID 07 (S1 Fig). In the same way the CD82-anchored sequence lacking the large extracellular loop (ΔLEL) was generated using the synthetic gene fragment SEQ ID 08, the restriction sites *Not*I/*Xho*I and the vector pCMV-CD63-mCherry-FLAG. This vector backbone was used to introduce retroviral ISU domain sequence elements SEQ IDs 9–12 (S1 Fig). The pCMVtag2B vector contains a neomycin selection marker. All constructs were verified by Sanger sequencing and restriction analysis.

The cloning sequence for the expression of the HHV6 protein was codon-optimised by the JAVA codon adaptation tool (JCAT) algorithm for E. coli expression (SEQ ID 13) and synthesised by ATGbiosynthetics (Merzhausen, Germany). The synthetic gene sequences were cloned into the expression vector pet16b (Novagen, Madison, WI, USA) using the restriction enzymes NdeI and XhoI (New England Biolabs). The cloned sequences were confirmed by Sanger sequencing.

BL21 *E*. *coli* competent cells (New England Biolabs) were transformed with the vector pet16B. The expression and isolation was performed similar as previously described [71]. The protocol was slightly changed and the overnight culture was diluted to an optical density at 600 nm wavelength (OD_600_) of 0.1 in 2 L 2YT-Medium. *E*. *coli* culture was shaken for 5 hrs without IPTG induction. All other steps were performed as previously described [72]. The fractions were separated by TGX Gels (BioRad, Munich, Germany) and stained with Coomassie blue solution (0.1% Coomassie Blue R250, 10% acetic acid, 40% methanol).

### Cell cultivation and transfection

HeLa (ATCC CCL2) or HEK293T (CRL-3216) cells were grown in Dulbecco’s modified Eagle’s medium (DMEM) supplemented with 10% fetal calf serum (FCS, Biochrom AG, Berlin, Germany), 100 IU/mL penicillin, 100 μg/mL streptomycin (PAA Laboratories, Cölbe, Germany), and 2 mM L-glutamine (Biochrom AG). FCS had been tested and was shown not to induce IL-10 in an assay described below. Cells were trypsinised and seeded in 6, 96 well plates (TPP, Munich, Germany) or for cLSM analysis in ibiTreat 8-well slides (IBIDI, Munich, Germany). After 24 h cells were transfected with the corresponding vector construct expressing the ISU domain and pCMV-CD82-YFP as control adding 2 μl of MetafectenePro (Biontex, Munich, Germany per well of a 96 well plate/ ibiTreat 8-well slides containing 2 x10^4^ Hela cells one day before transfection or 20 μl of MetafectenePro per well of a 6 well plate containing 0.5 x10^6^ HEK293T or HeLa cells. Transfection procedures were done at a confluency of approx. 80%. To prevent mycoplasma contamination cells were cultivated with DMEM medium supplemented with 10% FCS (Biochrom AG, Berlin, Germany) and an antibiotic mix containing tylosine 40 μg/ml (Sigma Aldrich, Steinheim, Germany), ciprofloxacin 20 μg/ml (Sigma Aldrich, Steinheim, Germany), and gentamycin 20 μg/ml (Applichem, Darmstadt, Germany) as well as 2 mM L-glutamine (Biochrom AG) until co-cultivation with PBMCs. All cells were maintained in an incubator at 37°C, 95% relative humidity and 5% CO_2_. All cells were mycoplasma free after tests described below.

### Analysis of protein expression by confocal laser scanning microscopy (cLSM)

Cells were fixed 24 hrs after transfection with 2% paraformaldehyde dissolved in PBS for 20 min, then washed with PBS and leaved in 200 μl 1xPBS + 20 μl Duolink mounting medium (Sigma Aldrich, Germany). Fixed cells were Images were obtained using a Zeiss LSM 780 confocal laser scanning inverted microscope (Carl Zeiss, Oberkochen, Germany) using a 63× oil immersion objective. The fluorescently-tagged proteins were detected employing a ZEISS ZEN smart set up instrument using the settings for 4,6-diamidino-2-phenylindole (DAPI), YFP and mCherry.

### Analysis of secreted proteins by Western blotting, ELISA and enhanced chemiluminescence

Secreted poly-histidine tagged proteins were isolated from pooled cell culture supernatants after centrifugation for 10 min at 2000 rpm in order to remove cell debris. The supernatants were incubated with 50 μl Dynabeads His-Tag (Invitrogen), incubated for 20 min at room temperature and washed the Dynabeads following manufactures instructions. After elution of poly-histidine tagged proteins with 50 μl His-elution buffer (300 mM imidazole, 50 mM sodium phosphate pH 8.0, 300 mM NaCl, 0.01% Tween-20), 50 μl of Laemmli buffer 2X were added and a SDS-PAGE using TGX gels (BioRad, Munich, Germany) was performed. The proteins were transferred onto Immobilon-P PVDF membranes (Millipore, Schwalbach, Germany). Blots were probed with 0.6 μg/mL of mouse anti-V5 antibodies labelled with horseradish peroxidase (HRP) (Invitrogene) or 0.5 μg/mL mouse anti-mCherry antibodies (Abcam, Cambridge, UK) and visualised with Pierce ECL Western blotting substrate (Thermo Scientific, Bonn, Germany). Western blot images were acquired using a Chemocam device (Intas, Göttingen, Germany) with implemented contrast correction.

Quantification of the secreted proteins was performed using the HHV6-derived protein (fraction B6, Panel A in S2 Fig) as standard for an ELISA. Ni(2+) chelate-coated 96 well plates (Pierce™ Nickel Coated Plates, Clear, Thermo Scientific, Dreieich, Germany) were pre-blocked with BSA and incubated with 50 μl of 40 x concentrated by ultrafiltration (Vivaspin20 columns, Sartorius, Göttingen, Germany) cell culture supernatant and 50 μl of the HHV6 protein (1:100 diluted in PBS) for 1 hr. Wells were washed three times with 200 μl of PBS/0.05% Tween-20, 100 μl of goat anti-6xHis-HRP antibody (Cat. No. 1269, Cambridge, United Kingdom) diluted 1:5000 in 5% BSA/PBS per well was added and incubated for 1 hour at room temperature. After washing five times with 200 μl of PBS/0.05% Tween20 the absorbance was determined.

### Isolation of human PBMCs

PBMCs were isolated from whole blood of healthy donors by Ficoll-Hypaque (PAA Laboratories, Austria) density centrifugation using Leucosep tubes (Greiner, Germany). PBMCs were cultivated at 37°C in RPMI 1640 with 10% FCS (Biochrome AG, Berlin, Germany) which had been selected for very low induction of IL-10 in normal human PBMCs.

### IL-10 assay

2 x10^4^ Hela cells were seeded per well of an 96 well plate and grown until the cells reached a confluency of approximately 80%. Cells were transfected with one of the plasmid DNA and 2 μl per well with MetafectenePro (Biontex, Munich, Germany). Cells were washed 3 times and co-cultivated with 3x10^5^ PBMCs per well overnight. In case of testing concentrated supernatant we incubated 105 ng of His tagged retroviral protein (dilutions in 200 μl media) with 3x10^5^ PBMCs per well. On the next day 100 μl of the supernatants were collected, centrifuged at 2000 g for 10 min and IL-10 secretion was tested by ELISA (BD Biosciences, San Diego, USA) technique.

### ELISA

96 wells plates (Nunc MaxiSorp, Thermo scientific, Germany) were coated with 100 μl of 1 μM synthetic peptide Biot-Ahx-Ahx-KQLQARILAVERYLKDQQL (Genaxxon, Germany) in carbonate buffer (15 mM Na_2_CO_3_, 35 mM NaHCO_3_, 0.2 g/L NaN_3_, pH 9.6) by overnight incubation at 4°C. The plates were washed three times with 300 μl/well wash buffer (1xPBS containing 0.05% Tween 20) and blocked for 2 hrs with 200 μl blocking buffer (3% bovine serum albumin in 1xPBS). Blocking buffer was replaced by 100 μl a dilution of purified IgG of the monoclonal antibody B3 obtained by immunisation of a mouse with an conjugate of the ISU peptide to bovine serum albumin (BSA). Primary IgG incubated for 1 hr and wells were washed 5 times with wash buffer. The primary antibody was detected by 100 μl/per well of 0.43 μg/ml anti-mouse HRP (DAKO, Denmark). After 1 hr plates were washed with 300 μl/per well washing buffer 5 times. Parallel in a 96 well plate (tissue culture test plate 96F, TPP, Switzerland) 2 x 10^4^ HeLa cells were seeded and transfected at confluency of 80% with the tANCHOR construct pCMV-CD82ΔLEL-HIV WT-mCherry (0.6 μg plasmid DNA per well, 2 μl MetafectenePro) for expressing the wt ISU domain of HIV-1. Non-transfected HeLa cells served as control. After 24 hrs Hela cells were fixed with 2% paraformaldehyde in 1xPBS for 30 min and washed two times with 300 μl of 1xPBS. Hela cells were blocked for 2 hrs with 200 μl blocking buffer (3% bovine serum albumin in 1xPBS), all further steps were performed as described for the peptide ELISA. The secondary antibody was detected by adding 100 μl of TMB (3,3',5,5'-tetramethylbenzidine) substrate (TMB substrate kit, Thermo Fisher) into each well and incubated at room temperature in dark for 15–30 min. After blue color development 100 μL of stop solution (0.16M H_2_SO_4_) was added. The absorbance of all wells was measured at 450 nm using a microplate reader (Multiscan^TM^GO, Thermo Scientific).

### LAL-test

The level of endotoxin was measured using the LAL-test kit from Thermofisher (Thermo Scientific, Braunschweig, Germany).

### Mycoplasma testing

Hela and HEK293T cells were tested for mycoplasma contamination (GATC Biotech, Konstanz, Germany) using the service MYCOPLASMACHECK.

### Ethical statement

The use of human blood has been approved by the ethical commission at the Medical Faculty of the Humboldt University Berlin. Written informed consent was provided by study participants.

## Supporting information

S1 Fig Nucleotide sequences of the TM domains containing the ISU domain of all pOUT and tANCHOR constructs used. The accession numbers of the sequences are displayed in the section material and methods.
(DOCX)

S2 Fig Characterisation of the HIS tagged gB protein of HHV-6. **(A)** Coomassie blue stained gel electrophoresis of the HHV-6 protein which was expressed and purified using His tag affinity chromatography, Fraction B6 was identified as pure protein, **(B)** fraction B6 was used for a colour response standard curve in order to calculate the amount of His tagged proteins.
(TIF)

S3 Fig Evidence that the expression of the WT and MUT HIV-1 and the WT and MUT PERV constructs was identical. Fluorescence (left column) and bright field (right column) microscopy image of HeLa cells expressing the CD82 anchored retroviral protein sequences tagged C-terminally with mCherry.
(TIF)

S4 Fig **(A) Differences in the release of IL-10 from PCMCs of six different donors.** The PBMCs of six different donors were incubated with the same amount of LPS (10 ng/ml) and the IL-10 release value was measured in an ELISA. **(B) Dose dependence of IL-10 induction by LPS. (C, D) Analysis of IL-10 release adding different amounts of plasmid.** Different amounts of plasmids encoding (C) tANCHOR and (D) pOUT were added. All measurements were performed in triplicates. The calculated p-values for the difference between the IL-10 release pf PBMCs incubated with untreated HeLa cells or with HeLa cells expressing the tANCHOR constructs with wt oder mut sequences 5.81E-12.
(TIF)
