# Supplementary material for: Absence of IL-10 production by human PBMCs co-cultivated with human cells expressing or secreting retroviral immunosuppressive domains
Source: PLoS One. 2018 Jul 12;13(7):e0200570. doi: 10.1371/journal.pone.0200570 (PMC6042780; doi:10.1371/journal.pone.0200570)
Supplement: S1 Fig — The accession numbers of the sequences are displayed in the section material and methods. (DOCX) [file pone.0200570.s001.docx]

| **SEQ ID 01 (HIV WT pout)** |
| --- |
| ccatgggagtgaaagttctttttgcccttatttgtattgctgtggccgaggccgaattccaattgCTGACGGTACAGGCCAGACAATTATTGTCTGATATAGTGCAGCAGCAGAACAATTTGCTGAGGGCTATTGAGGCGCAACAGCATCTGTTGCAACTCACAGTCTGGGGCATCAAACAGCTCCAGGCAAGAATCCTGGCTGTGGAAAGATACCTAAAGGATCAACAGCTCCTGGGGATTTGGGGTTGCTCTGGAAAACTCATTTGCACCACTGCTGTGCCTTGGAATGCTAGTTGGAGTAATAAATCTCTGGAACAGATTTGGAATAACATGACCTGGATGGAGTGGGACAGAGAAATTAACAATTACACAAGCTTAATACACTCCTTAATTGAAGAATCGCAAAACCAGCAAGAAAAGAATGAACAAGAATTATTGGAATTAGATAAATGGGCAAGTTTGTGGAATTGGTTTAACatcgattttggtaagcctatccctaaccctctcctcggtctcgattctacgcccgggcatcatcatcatcatcattaacatatg |
| **SEQ ID 02 (HIV MUT pout)** |
| ccatgggagtgaaagttctttttgcccttatttgtattgctgtggccgaggccgaattccaattgCTGACGGTACAGGCCAGACAATTATTGTCTGATATAGTGCAGCAGCAGAACAATTTGCTGAGGGCTATTGAGGCGCAACAGCATCTGTTGCAACTCACAGTCTGGGGCATCAAACAGGGCGGAGGAGGCATCCTGGCTGTGGAAAGAGGTCTAAAGGGCCAACAGCTCCTGGGGGGTTGGGGTTGCTCTGGAAAACTCATTTGCACCACTGCTGTGCCTTGGAATGCTAGTTGGAGTAATAAATCTCTGGAACAGATTTGGAATAACATGACCTGGATGGAGTGGGACAGAGAAATTAACAATTACACAAGCTTAATACACTCCTTAATTGAAGAATCGCAAAACCAGCAAGAAAAGAATGAACAAGAATTATTGGAATTAGATAAATGGGCAAGTTTGTGGAATTGGTTTAACatcgattttggtaagcctatccctaaccctctcctcggtctcgattctacgcccgggcatcatcatcatcatcattaacatatg |
| **SEQ ID 03 (PERV WT pout)** |
| CCATGGGAGTGAAAGTTCTTTTTGCCCTTATTTGTATTGCTGTGGCCGAGGCCGAATTCCAATTGCTTATCACTGGACCTCAGCAATTAGAGAAAGGCCTGAGTAATCTGCACCGGATCGTGACCGAGGACCTGCAAGCGCTGGAAAAAAGCGTATCGAATTTGGAAGAGTCTCTGACAAGCCTGTCAGAAGTCGTTCTGCAGAATCGCCGCGGACTGGACTTACTGTTCCTGAAAGAGGGCGGCTTGTGTGTCGCCCTTAAAGAAGAGTGCTGCTTTTATGTGGACCATAGCGGCGCCATCCGTGATTCCATGAGCAAACTGCGTGAACGCCTCGAACGACGCCGCCGCGAGCGTGAAGCCGATCAAGGCTGGTTTGAAGGCTGGTTCAATATCGATTTTGGTAAGCCTATCCCTAACCCTCTCCTCGGTCTCGATTCTACGCCCGGGCATCATCATCATCATCATTAACATATG |
| **SEQ ID 04 (PERV MUT pout)** |
| CCATGGGAGTGAAAGTTCTTTTTGCCCTTATTTGTATTGCTGTGGCCGAGGCCGAATTCCAATTGCTTATCACTGGACCTCAGCAATTAGAGAAAGGCCTGAGTAATCTGCACCGGATCGTGACCGAGGACCTGCAAGCGCTGGAAAAAAGCGTATCGAATTTGGAAGAGTCTCTGACAAGCCTGTCAGAAGTCGTTGGAGGTGGTGGACGCGGACTGGACTTACTGGGACTGAAAGGAGGCGGCTTGTGTGTCGGTCTTAAAGAAGAGTGCTGCTTTTATGTGGACCATAGCGGCGCCATCCGTGATTCCATGAGCAAACTGCGTGAACGCCTCGAACGACGCCGCCGCGAGCGTGAAGCCGATCAAGGCTGGTTTGAAGGCTGGTTCAATATCGATTTTGGTAAGCCTATCCCTAACCCTCTCCTCGGTCTCGATTCTACGCCCGGGCATCATCATCATCATCATTAACATATG |
| **SEQ ID 05 (Mulv WT pout)** |
| CCATGGGAGTGAAAGTTCTTTTTGCCCTTATTTGTATTGCTGTGGCCGAGGCCGAATTCCAATTGACTACCGCCCTGGTCGCCACCCAGCAGTTTCAGCAGCTCCATGCTGCCGTACAAGATGATCTCAAAGAAGTCGAAAAGTCAATTACTAACCTAGAAAAGTCTCTTACTTCGTTGTCTGAGGTTGTACTGCAGAATCGACGAGGCCTAGACCTGTTGTTCCTAAAAGAGGGAGGACTGTGTGCTGCCCTAAAAGAAGAATGTTGTTTCTATGCTGACCATACAGGCCTAGTAAGAGATAGTATGGCCAAATTAAGAGAGAGACTCTCTCAGAGACAAAAACTATTTGAGTCGAGCCAAGGATGGTTCGAAGGATGGTTTAACAGATCCATCGATTTTGGTAAGCCTATCCCTAACCCTCTCCTCGGTCTCGATTCTACGCCCGGGCATCATCATCATCATCATTAACATATG |
| **SEQ ID 06 (Mulv MUT pout)** |
| CCATGGGAGTGAAAGTTCTTTTTGCCCTTATTTGTATTGCTGTGGCCGAGGCCGAATTCCAATTGACTACCGCCCTGGTCGCCACCCAGCAGTTTCAGCAGCTCCATGCTGCCGTACAAGATGATCTCAAAGAAGTCGAAAAGTCAATTACTAACCTAGAAAAGTCTCTTACTTCGTTGTCTGAGGTTGTACTGCAGAATCGACGAGGCCTAGACCTGTTGTTCCTAAAACGAGGAGGACTGTGTGCTTTTCTAAAAGAAGAATGTTGTTTCTATGCTGACCATACAGGCCTAGTAAGAGATAGTATGGCCAAATTAAGAGAGAGACTCTCTCAGAGACAAAAACTATTTGAGTCGAGCCAAGGATGGTTCGAAGGATGGTTTAACAGATCCATCGATTTTGGTAAGCCTATCCCTAACCCTCTCCTCGGTCTCGATTCTACGCCCGGGCATCATCATCATCATCATTAACATATG |
| **SEQ ID 07 (CD82)** |
| gctagcgcggccgccaccatggattacaaggatgacgacgataagagcccgggcggatccatgggctcagcctgtatcaaagtcaccaaatactttctcttcctcttcaacttgatcttctttatcctgggcgcagtgatcctgggcttcggggtgtggatcctggccgacaagagcagtttcatctctgtcctgcaaacctcctccagctcgcttaggatgggggcctatgtcttcatcggcgtgggggcagtcactatgctcatgggcttcctgggctgcatcggcgccgtcaacgaggtccgctgcctgctggggctgtactttgctttcctgctcctgatcctcattgcccaggtgacggccggggcactcttctacttcaacatgggcaagctgaagcaggagatgggtggcatcgtgactgagctcattcgagactacaacagcagtcgcgaggacagcctgcaggatgcctgggactacgtgcaggctcaggtgaagtgctgcggctgggtcagcttctacaactggacagacaacgctgagctcatgaatcgccctgaggtcacctacccctgttcctgcgaagtcaagggggaagaggacaacagcctttctgtgaggaagggcttctgcgaggcccccggcaacaggacccagagtggcaaccaccctgaggactggcctgtgtaccaggagggctgcatggagaaggtgcaggcgtggctgcaggagaacctgggcatcatcctcggcgtgggcgtgggtgtggccatggtcgagctcctggggatggtcctgtccatctgcttgtgccggcacgtgcattccgaagactacagcaaggtccccaagtacaagcttatcgataccgtcgacctcgag |
| **SEQ ID 08 (CD82ΔLEL)** |
| gctagcgcggccgccaccatggattacaaggatgacgacgataagagcccgggcggatccatgggctcagcctgtatcaaagtcaccaaatactttctcttcctcttcaacttgatcttctttatcctgggcgcagtgatcctgggcttcggggtgtggatcctggccgacaagagcagtttcatctctgtcctgcaaacctcctccagctcgcttaggatgggggcctatgtcttcatcggcgtgggggcagtcactatgctcatgggcttcctgggctgcatcggcgccgtcaacgaggtccgctgcctgctggggctgtactttgctttcctgctcctgatcctcattgcccaggtgacggccggggcactcttctacttcaacatgggcaagctgaagcaggaattcggaaagggcccgcggttcgaaggtaagcctatccctaaccctctcctcggtctcgattctacgcgtaccggtcatcatcaccatcaccatgatatcggaggaggaggagtgcaggcgtggctgcaggagaacctgggcatcatcctcggcgtgggcgtgggtgtggccatggtcgagctcctggggatggtcctgtccatctgcttgtgccggcacgtgcattccgaagactacagcaaggtccccaagtacaagcttatcgataccgtcgacctcgag |
| **SEQ ID 09 (HIV WT tANCHOR)** |
| GCGGCCGCGAATTCCTGCTGTTGCAACTCACAGTCTGGGGCATCAAACAGGGTGGCGGtGGAATCCTGGCTGTGGAAAGAGGTCTAAAGGGTCAACAGCTCCTGGGGGGTTGGGGTTGCTCTGGAAAACTCATTTGCACCACTGCTGTGCCTTGGAATGATATCTTTGCGGCCGCAAGCTTG |
| **SEQ ID 010 (HIV MUT tANCHOR)** |
| GCGGCCGCGAATTCCTGCTGTTGCAACTCACAGTCTGGGGCATCAAACAGGGTGGCGGtGGAATCCTGGCTGTGGAAAGAGGTCTAAAGGGTCAACAGCTCCTGGGGGGTTGGGGTTGCTCTGGAAAACTCATTTGCACCACTGCTGTGCCTTGGAATGATATCTTTGCGGCCGCAAGCTTG |
| **SEQ ID 11 (PERV WT tANCHOR)** |
| GCGGCCGCGAATTCCTGCTGTTGCAACTCACAGTCTGGGGCATCAAACAGGGTGGCGGtGGAATCCTGGCTGTGGAAAGAGGTCTAAAGGGTCAACAGCTCCTGGGGGGTTGGGGTTGCTCTGGAAAACTCATTTGCACCACTGCTGTGCCTTGGAATGATATCTTTGCGGCCGCAAGCTTG |
| **SEQ ID 12 (PERV MUT tANCHOR)** |
| GaattcattgtaacggaagatctccaagccctagaaaaatctgtcagtaacctggaggaatccctaacctccttatctgaagtggttGGAGGTGGAGGAagggggttagatctgttaGGTctaaaaGGAggagggttatgtgtaggattaaaagaggaatgctgcttctatgtagatcactcaggagccgatATC |
