# Supplementary figures and images for: Absence of IL-10 production by human PBMCs co-cultivated with human cells expressing or secreting retroviral immunosuppressive domains
Source: PLoS One. 2018 Jul 12;13(7):e0200570. doi: 10.1371/journal.pone.0200570 (PMC6042780; doi:10.1371/journal.pone.0200570)

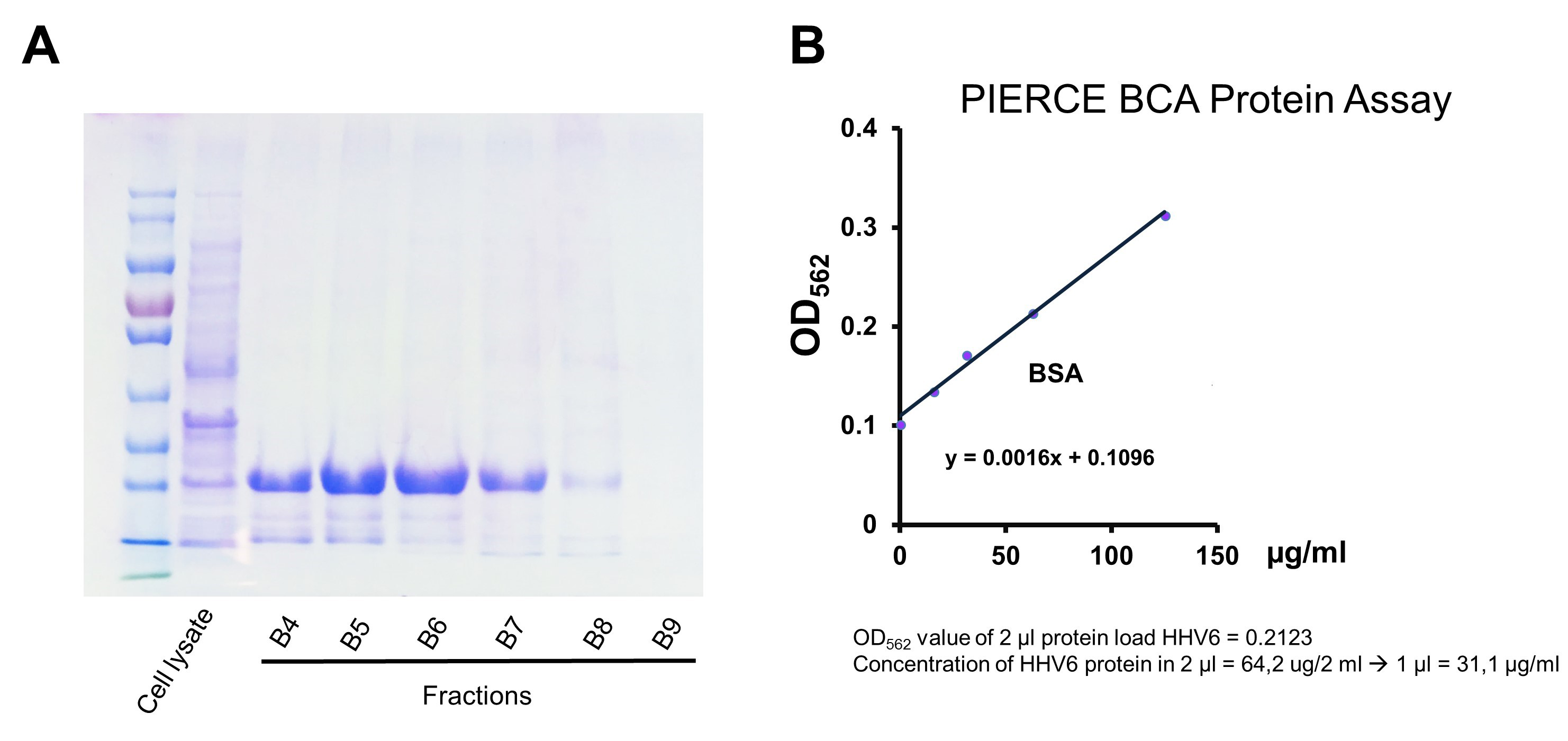

Supplement: S2 Fig — (A) Coomassie blue stained gel electrophoresis of the HHV-6 protein which was expressed and purified using His tag affinity chromatography, Fraction B6 was identified as pure protein, (B) fraction B6 was used for a colour response standard curve in order to calculate the amount of His tagged proteins. (TIF) [file pone.0200570.s002.tif]

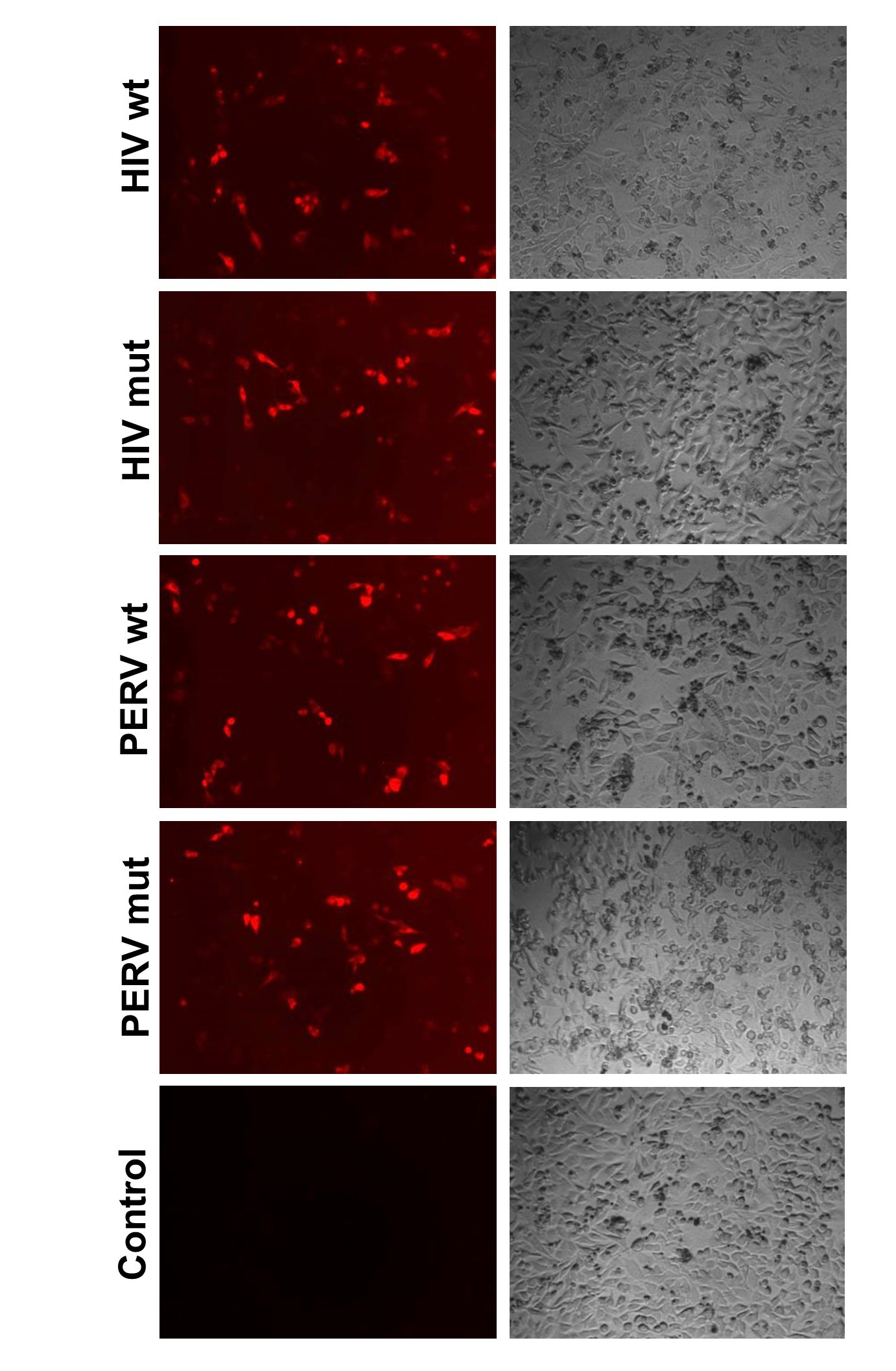

Supplement: S3 Fig — Fluorescence (left column) and bright field (right column) microscopy image of HeLa cells expressing the CD82 anchored retroviral protein sequences tagged C-terminally with mCherry. (TIF) [file pone.0200570.s003.tif]

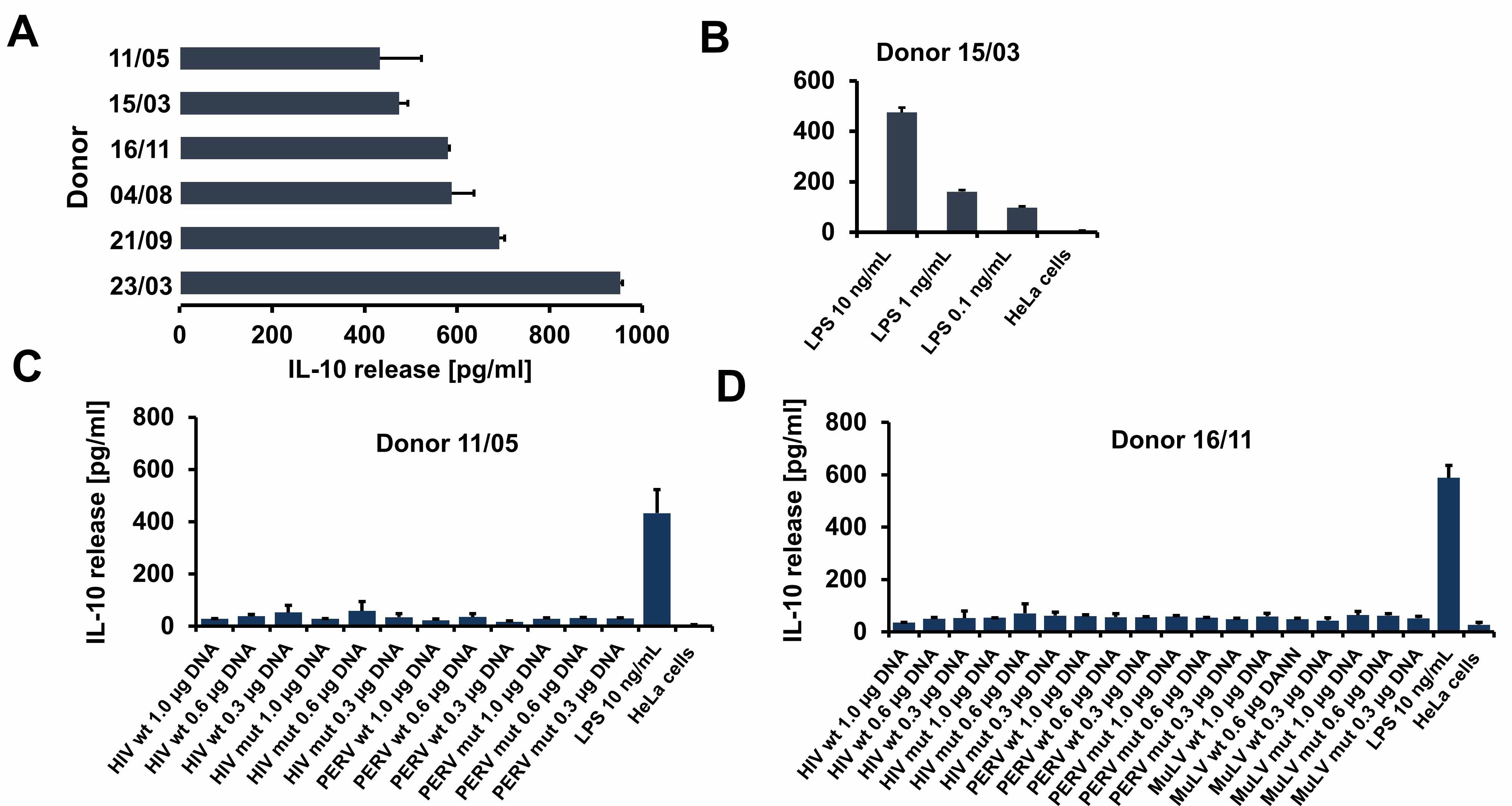

Supplement: S4 Fig — (A) Differences in the release of IL-10 from PCMCs of six different donors. The PBMCs of six different donors were incubated with the same amount of LPS (10 ng/ml) and the IL-10 release value was measured in an ELISA. (B) Dose dependence of IL-10 induction by LPS. (C, D) Analysis of IL-10 release adding different amounts of plasmid. Different amounts of plasmids encoding (C) tANCHOR and (D) pOUT were added. All measurements were performed in triplicates. The calculated p-values for the difference between the IL-10 release pf PBMCs incubated with untreated HeLa cells or with HeLa cells expressing the tANCHOR constructs with wt oder mut sequences 5.81E-12. (TIF) [file pone.0200570.s004.tif]
